# Seroprevalence of HBV and HCV in primary hepatocellular carcinoma patients in Zimbabwe

**DOI:** 10.1186/1750-9378-4-15

**Published:** 2009-10-08

**Authors:** Nyasha Chin'ombe, Evans Chavhunduka, Hilda T Matarira

**Affiliations:** 1 University of Cape Town, Faculty of Health Sciences, Department of Clinical Laboratory Sciences, Division of Medical Virology, Observatory 7925, Cape Town, South Africa; 2 University of Zimbabwe, Faculty of Health Sciences, Department of Chemical Pathology, PO Box A178 Avondale, Harare, Zimbabwe; 3 University of Zimbabwe, Faculty of Veterinary Sciences, PO Box MP167 Mount Pleasant, Harare, Zimbabwe; 4 Great Zimbabwe University, Box 1460, Masvingo, Zimbabwe

## Abstract

**Background:**

Primary hepatocellular carcinoma (PHC) is one of the most common cancers in Zimbabwe. Hepatitis B virus (HBV) and hepatitis C virus (HCV) are suspected to play a major role in causing this cancer. The objective of this study was to determine the seroprevalence of HBV and HCV in PHC at Parirenyatwa Referral Hospital in Zimbabwe. We evaluated the serological markers of the two viruses in patients with PHC using commercially available enzyme-linked immunosorbent kits.

**Results:**

Out of the 60 patients with PHC, 48.3% were seropositive for HBV and 20.0% were seropositive for HCV. Co-infection by HCV and HBV was found in 8% of the patients. Only 13.3% of the health controls (blood donors) were positive for HBV. All the controls were negative for HCV.

**Conclusion:**

The high seropositivity of HBV and HCV in PHC in Zimbabwe suggested that the two viruses were a major cause of the cancer.

## Background

Liver cancer is one of the most common cancers in Zimbabwe and Africa [1,2]. Hepatitis B virus (HBV) and hepatitis C virus (HCV) are suspected to play an important aetiological role in liver cancer in this region and the continent. HBV is a partially double-stranded DNA virus and is an important cause of morbidity and mortality worldwide [3,4]. Globally, it is estimated that about 2 billion people have been infected with HBV and about 350 million people are chronically infected [5]. Most of the chronically-infected people are found in developing countries and are at a high-risk of developing liver cirrhosis and hepatocellular carcinoma [6,7]. Areas with high prevalence of HBV include Southeast Asia, China and Africa, where approximately 10% of the population are chronic carriers [5,8]. In persons infected with HBV, morbidity and mortality result when inflammatory liver disease progresses to cirrhosis and primary hepatocellular carcinoma [8]. High-risk groups of HBV infection include intravenous drug users, attendees of sexually transmitted disease clinics, homosexual men, patients undergoing haemodialysis, children born to mothers who are hepatitis B surface antigen positive and health care workers [6,7,9].

HCV is a positive-stranded RNA virus which belongs to the *Flaviviridae *family [10]. Worldwide, the virus infects about 170 million people [11]. Infection by this virus can be resolved spontaneously in only about 15-20% of cases and chronic infection is established in the remaining 80-85% [12]. Most of these chronic HCV infections usually lead to severe hepatitis, liver cirrhosis and hepatocellular carcinoma [13-17]. The risk of developing PHC ranges from 1-5% after about two decades of chronic infection [18]. HCV infection usually occurs following direct parenteral exposure to contaminated body fluids, especially blood [16,19]. The prevalence of both HBV and HCV in PHC in Zimbabwe is poorly known. It was against this background that we set out to evaluate the presence of antibodies to HBV surface antigen and HCV in patients with PHC at Parirenyatwa Central Hospital in Zimbabwe.

## Results

### Patient data

Data from hospital records showed that all the patients presented with abdominal distending discomfort, characterised by right upper quadrant pain and hepatic mass enlargement in some cases. Weight loss was observed in all the patients with almost a third showing that they were jaundiced. Ascites and fever symptoms were not uncommon in the patients on presentation or during hospitalisation. All the patients had impaired liver function in one or more of serum aminotransferases, prolonged prothrombin time and albumin levels. Out of the 60 patients, 53 (88%) had serum gamma-glutamyl transferase (GGT) levels above the reference range, with 10 of them having GGT values greater than 200 IU/L. Of all the patients, 30 (50%) had alanine transaminase (ALT) levels elevated above the upper limit of normal range. The age ranges of patients and controls are given in Table 1.

**Table 1:** Sex and age parameters for PHC patients and health controls

**Parameter**	**PHC patients (n = 60)**	**Health Controls (n = 30)**
Age range	16 - 81 years	16 - 69 years
Mean age	45.7 years	38.5 years
Male to Female ratio	2:1	2.08:1

The clinical management of the patients was also assessed from the records. Most of the patients were of poor social background, either in the lowest income group or not employed at all. Some of the males (n = 11) over 30 years of age had a history of alcohol drinking. Of all the patients, 7 (12%) had cirrhosis clearly indicated in their records. Alpha-fetoprotein (AFP) levels were greater than 400 ng/ml in 33 patients (55%) and 21 patients (35%) with AFPs less than 400 ng/ml had confirmation of PHC by ultrasound and cytology. The difference in age distribution in PHC patients and health individual controls was not significant (p > 0.05). The difference in sex between the two groups was also not significantly different (p > 0.05).

### HBsAg and HCV antibody status in PHC patients

The seroprevalence of HBV and HCV in patients with PHC and health blood donours is summarized in Table 2. Of the 60 PHC cases, 29 patients (48%) were positive for HBsAg of which 7 were females and 22 were males. The age range of patients was 18-70 years and the mean was 43.8 years. Only 10 (34%) of the patients were over 45 years of age. The mean age of HBsAg negative patients was 47.6 years. There was no significant discrepancy between the mean ages of HBsAg positive and negative patients. Only 4 control subjects were positive for HBsAg. The relative risk for developing PHC in patients with HBsAg was high when control subjects were considered as a reference group (odds ratio = 1.62, 95% CI 0.69-1.64).

**Table 2:** Seropositivity and negativity for Anti-HCV and HBsAg in PHC and health blood donors

**Parameter**	**PHC (n = 60)**	**Control s (n = 30)**	**p value**
Anti-HCV positivity	12 (20%)	0 (0%)	<0.05
Anti-HCV negativity	48 (80%	30 (100%)	<0.05
HBsAg positivity	29 (48.3%)	4 (13.3%)	<0.05
HBsAg negativity	31 (51.7%)	26 (86.7%)	<0.05
Dual anti-HCV & HBsAg positivity	5 (8.3%)	0 (0%)	-
Anti-HCV and/or HBsAg positivity	36 (60%)	4 (13.3%)	-
Anti-HCV and/or HBsAg negativity	24 (40%)	26 (86.7%)	-

A total of 12 (20%) PHC patients were positive for anti-HCV and 8 (67%) of these patients were over 45 years of age (66.7%) and the other remaining 4 (33%) were below 45 years. All the control patients from blood donors were negative for anti-HCV antibodies. The difference in males and females infected with HCV was not significant (p > 0.05). The mean age of patients who were positive for anti-HCV was 51.26 years and the age range was 16-70 years. The odds ratio or relative risk for the development of PHC in anti-HCV positive patients could not be ascertained because none of the control individuals was positive and the calculation required an integer for the reference sample. Of the 12 patients who were anti-HCV-positive, 4 had AFP levels greater than 400 ng/ml whilst two had their AFP levels within the normal range (0-10 ng/ml). The prevalence of HBsAg was significantly higher than that of anti- HCV both in PHC patients and controls (p < 0.05). The presence of both anti-HCV and HBsAg was noted in 5 patients. It was also noted that more than half of the patients had infection with HBV and/or HCV.

The relative risk of developing PHC was strongly associated with HBV infection. The unadjusted odds ratio for PHC development was high (1.615; 95% CI 0.69 - 1.65) for overall HBV infection. For those patients with HBsAg alone, the risk for PHC development, using the cases which were negative for HBsAg as a reference group and healthy individuals as controls, was still high (odds ratio = 1.48: 95% CI 0.81-1.53). The relative risk for PHC developing in dual infection with HCV using non-HBsAg cases as references and healthy individuals as controls, was low (odds ratio = 0.818). The odds ratio in patients positive for either anti-HCV and HBsAg or both was even higher (odds ratio = 1.875; 95% CI 0.54 - 1.88).

## Discussion

Parirenyatwa Hospital is a supra-referral hospital in Zimbabwe. Patients suspected to have primary hepatocellular carcinoma are referred from primary hospitals and clinics around the country to this referral hospital. Therefore, although our study had only 60 PHC patients, they were likely to be representative of the whole country. This study strongly suggested that Hepatitis B and C viruses were important etiological factors of PHC in Zimbabwe. Although other factors such as exposure to aflatoxins (from mouldy grains and cereals) and alcohol consumption were believed to be important, hospital records for most of the patients lacked the information. Alcohol consumption was documented in only 20% of the patients. Information on risks to HBV and HCV infection such as history of blood transfusion, use of intravenous drug abuse and traditional surgical procedures could also not be obtained from the files. These parameters could have some influence on the prevalence of seropositivity of PHC patients to HBV and HCV. Aminotrasferase levels were assessed in the records and most patients had abnormal levels of one or more of ALT, AST and GGT being elevated. This is in agreement with studies that showed extensive hepatocellular damage in PHC patients [20-23]. All the patients who were positive for anti-HCV and HBsAg had elevated hepatic enzymes. Albumin was low and prothrombin time was prolonged in most of the cases. Hepatic aminotransferases were not measured in the controls to evaluate hepatocyte damage in those who were HBsAg-positive. There is a strong link between HCV and cirrhosis with 60-80% of HCV-positive PHC patients being cirrhotic [24-26]. It could have been interesting to note this association in cirrhotic HCV positive PHC cases. Only seven of the patients whose cirrhotic condition was well documented were in the study and anti-HCV antibodies were only found in two patients who also had HBsAg in their sera. The cirrhotic condition of other patients could not be established and hence an association with HCV could not be studied. The biopsy rate is low due to poor prognosis and late presentation. Only 6 patients had histologically verified PHC status. The diagnosis of PHC based on biochemical evaluation of AFP is controversial especially when levels less than 400 ng/ml are encountered. AFP levels were greater than 400 ng/ml in 33 patients, six had histological proof and the remainder were confirmed by cytology and ultrasound scanning when focal or multifocal lesions were suggested. The diagnosis techniques employed were convincing that the cases certainly had PHC.

The risks of acquiring HBV and HCV infection could not be assessed for this study. The detection of viral markers in two female teenagers with PHC suggested that they could have acquired the viruses parenterally or vertically. In previous studies of PHC, anti-HCV and HBsAg have been found in even younger patients of 16 years by biopsy [20,27]. The mean age of PHC patients of 45.7 years in our study agreed well with those found in studies in South Africa (45 years), Nigeria (46.7 years) and Southern Africa (44.8 years) [20,23,27]. In Mozambique the mean age in Shangaan ethnic group was 33.4 years and 50% of the patients were below 30 years [21].

The presence of antibodies to the HBsAg in some of the PHC patients clearly indicated HBV infection. The presence of HBsAg is normally used as a diagnostic marker to indicate acute or chronic infection [28]. It could have been interesting if we had also looked at the presence of HBV DNA in the samples. However, in highly endemic areas, HBV DNA has also been detected in blood or liver of patients without HBsAg [29]. The diagnosis of HBV by detection of HBsAg is very reliable only in acute phase infection and present infection [27,30]. It is not indicative of past infection. However most of the PHC studies done showed that HBsAg is expressed in 70-80% of HBV positive patients who were found to be positive for viral DNA [30,31]. The reliability of HBsAg as a marker of HBV infection in PHC is based on the expression of this antigen from the integrated viral genome in the hepatocyte DNA during vigorous active hepatocyte proliferation and turnover. At least 80% of HBsAg carriers have evidence of chromosomal integration of viral sequences, by Southern hydridisation of tumour DNA [31,32]. Other markers that show past infection could have been assayed for to evaluate overall exposure. Our results confirmed previous reports of high prevalence of HBsAg in patients with PHC in sub-Sahara Africa, especially the southern parts [20,27,33].

The presence of antibodies to HCV in some of the patients also suggested that the virus was also an important aetiological agent in PHC in Zimbabwe. It was not clear whether some of the patients with anti-HCV had already resolved the virus. The resolution of HCV infection occurs in 15-20% of patients [12,34]. It could have been an interesting study if we had also looked at the presence of HCV RNA in the samples. However, it takes between a decade and three for the development of PHC from time of infection with HCV and the viral RNA may disappear upon resolution but the antibodies persist [35,36]. Detection of viraemia by polymerase chain reaction (PCR) could also have been done to detect cases where there was active viral replication.

In our study, 48.3% of 60 PHC patients were HBsAg positive and 20% were anti-HCV positive. In a previous study in Zimbabwe, HBsAg was detected in 42.6% of the PHC patients of the 62 patients studied [33]. Positivity for anti-HCV was in 23.8% using a lesser sensitive and specific second generation assay [33]. The two studies show that HBV remains the major infection of hepatitis viruses in the region. The prevalence of HBsAg in controls (13.3%) was less than the prevalence of 24.2% observed in a study of 660 health individuals [37,38]. This probably was due to the smaller proportion of the present study group (30 blood donors). The observation of no anti-HCV positive cases in controls is agreement with data from the National Blood Transfusion Service which show a prevalence of less than 1% in healthy blood donors. However, another study in Zimbabwe has observed an anti-HCV seropositivity of 8% in healthy rural individuals [22]. Co-infection by HCV and HBV was found in this study. Although HBV and HCV may be independent risk factors of PHC, dual infection is likely to increase the risk of primary hepatocellular carcinogenesis. Some studies have also reported dual infection by HBV and HCV in PHC [20,23,30]. However, the absence of serological markers for either HBV and/or HCV in PHC in some PHC patients could not rule out the role of the two viruses in carcinogenesis. Studies have shown that hepatic tissues could have viral genomic sequences in one-third or more of the serologically negative subjects [38]. It was also suspected that aflatoxin contamination could also have played a significant role in PHC development since Zimbabwe has been found to have this post-harvest problem [39]. This study and previous ones, therefore, demonstrate that HBV and HCV are important factors in chronic liver disease and subsequently PHC development in Southern Africa.

## Conclusion

HBV and HCV play a significant role in the aetiology of hepatocellular carcinoma in Zimbabwe.

## Patients and Methods

### Study subjects

The sixty patients were presented to Parirenyatwa Hospital between October 1999 and August 2000 and were diagnosed as having PHC. Sera for the patients were collected from the Radioimmunoassay (RIA) Laboratory of the Public Health Laboratory (PHL). The serum samples were collected after determination of the levels of the tumour marker, Alpha-fetoprotein (AFP). The sera were stored at -20°C until assayed for anti-HCV antibodies and HBsAg. The medical records of the patients were also examined for diagnostic tests and evaluations suggesting or confirming primary hepatocellular carcinoma. The tests included ultrasound scanning, X-ray photography, needle aspirate cytological proof and liver biopsy histology. Of the 60 patients recruited in this study, only 6 had liver biopsy evaluation of PHC by histological examination. The rest of the patients' diagnosis depended on ultrasound scanning, cytology (fine needle aspirate), clinical features and biochemical evaluations of liver functions and AFP levels. Data on age and sex were also extracted. Controls (health blood donors) (n = 30) were included in the study. They were mainly health blood donors from the National Blood Transfusion Services.

### Laboratory tests of anti-HCV and anti-HBV serological markers

All samples (patients and controls) were tested for the anti-HCV using the commercially available MUREX anti-HCV (Version 4.0) kit (Abbott Diagnostic Division, RSA) according to manufacturer's instructions. The MUREX anti-HCV (Version 4.0) is a third generation solid phase enzyme immunoassay in which a mixture of highly purified viral antigens is coated into microwells of a microtitre plate. The antigens contain sequences from the core (Structural), NS3 protease/helicase (non-structural), NS4 (non-structural) and the NS5 replicase (non-structural) regions of HCV. During incubation with samples any anti-HCV antibodies present in the sample will bind to the immobilized antigens. Following washing to remove unbound material, the captured anti-HCV antibodies are incubated with peroxidase conjungated monoclonal antihuman IgG. The conjugate will bind to the immobilized anti-HCV through the anti-human IgG moiety. After removal of excess conjugate, bound enzyme is detected by the addition of a solution containing 3,3;5,5- tetramethyl benzidine (TMB) and hydrogen peroxide. The enzyme peroxidase changes the substrate to a purple colour in the wells, which contain anti-HCV positive samples. The enzymatic reaction is terminated with sulphuric acid to give an orange colour that is read spectrophotometrically. The amount of conjugate bound and hence colour in the wells, is directly proportional to the concentration of antibodies to HCV in the specimen.

All the samples were also tested for HBsAg using the Bioelisa Kit (Biokit, RSA) according to manufacturer's instructions. The Bioelisa Kit is an ELISA test kit for the detection of the hepatitis B surface antigen (HBsAg) in human sera or plasma. The bioelisa HBsAg assay is a direct immunoenzymatic method of the "sandwich" type in which guinea pig anti-HBs antibodies coated onto microtitre plate wells act as the capture antibody and goat anti-HBs anti-bodies marked with peroxidase serve as conjugate antibodies. During the test procedure, the sample to be analysed is incubated in one of the antibody coated wells. If the sample contains HBsAg, the antigen will bind to the antibody on the plate. After washing to eliminate any unbound material, goat anti-HBs conjugate is added to the well and allowed to react with the antigen-antibody complex formed in the first incubation. After a second incubation and subsequent washing, an enzyme substrate solution containing a chromogen (TMB) is added. This solution will develop a blue colour if the sample is HBsAg positive. The blue colour changes to yellow after blocking with sulphuric acid and can be read spectrophotometrically. The intensity of the colour is proportional to the amount of HBsAg in the test specimens.

The results were analysed. Continuous variables were compared using the Student's t-test. Categorical data were compared using the Pearson's Chi-squared test or Fisher's exact test. P-values < 0.05 were considered statistically significant. Statistical analyses were performed using the Epiinfo program, version 2000 package.

## Abbreviations

HBV: Hepatitis B virus; HBsAg: Hepatitis B surface antigen; HCV: Hepatitis C virus; PHC: primary hepatocellular carcinoma; AFP: alpha-fetoprotein; ALT: alanine transaminase; AST: aspartate transaminase; GGT: gamma glutamyl transpeptidase.

## Competing interests

The authors declare that they have no competing interests.

## Authors' contributions

NC and EC collected patient specimens, designed and performed the experiments and analyzed the data. HTM participated in the designing of the whole study. All the authors participated in the writing of this manuscript. All the authors read and approved the manuscript.
